# The Physiology of Mormonism

**Published:** 1863-07

**Authors:** Charles C. Furley

**Affiliations:** Assistant Surgeon United States Army


### THE PHYSIOLOGY OF MORMONISM.

By CHARLES C. FURLEY, M. D., Assistant Surgeon United States Army.

On a recent visit to Salt Lake I had good opportunities for observing and inquiring into the effects of polygamy, as practically exemplified in the case of that people. While sojourning there I mingled much amongst them, visiting them in their homes, and seeing them at their public assemblies and places of business and pleasure; wherefore, I feel qualified to speak of the results of their peculiar institutions, both in their social, physiological and intellectual bearings. It is, however, chiefly as a physiologist that I shall, at present, consider the subject, and, in this view, I must say, the consequences of the Mormon system, as we find them illustrated in the inhabitants of Salt Lake, are, in every aspect of the case, hurtful and degrading.

A marked physiological inferiority strikes the stranger, from the first, as being one of the characteristics of this people. A certain feebleness and emaciation of person is common amongst every class, age and sex; while the countenances of almost all are stamped with a mingled air of imbecility and brutal ferocity. This, in fact, is their true character; they being obsequious and yielding to their superiors—to strangers sullen and spiteful, while among themselves they are cold and unamiable. In the faces of nearly all, one detects the evidences of conscious degradation, or the bold and defiant look of habitual and hardened sensuality—the women, with but few exceptions, serinking from the gaze of the stranger, as if fully alive to the false and degraded position they are forced to occupy. Some seem overwhelmed with shame; others wear a forlorn and haggard appearance, while a few put on a cheerful air, affecting to be satisfied with their sad condition.

Without entering into minutiæ, I may instance the following as a few of the bodily peculiarities that strike the medical man in mingling with the inhabitants of Salt Lake City: Besides the attenuation mentioned, there is a general lack of color—the cheeks of all being sallow and cadaverous, indicating an absence of good health. The eye is dull and lustreless—the mouth almost invariably coarse and vulgar. In fact, the features—the countenance—the whole face, where the divinity of the man should shine out, is mean and sensual to the point of absolute ugliness. I have nowhere seen anything more pitiful than the faces of the women here, or more disgusting than the entire appearance of the men. It is a singular circumstance that the physiognomical appearance of the children are almost identical. The striking peculiarity of the facial expression—the albuminous types of constitution, the light yellowish hair, the blue eye and the dirty, waxen hue of the skin, indicate plainly the diathesis to which they belong. They are puny and of a scorbutic tendency. The external evidences are numerous that these polygamic children are doomed to an early death—the tendency to phthisis pulmonials being eminent and noticeable.

The evidences of natural degeneracy are more palpable in the youthful than in the adult population; the evils of this pernicious system not having taken full effect upon the latter.

A more feeble and ill-looking race of children I have not met with, even among the vice and squalor of our larger cities. One looks in vain for those signs of constitutional vigor and sturdy health common to the juvenile portion of what may be considered but a country town. So far as food, climate and other external causes are concerned, the children, as well as the adults here, are favorably circumstanced; their sanitary conditions are generally good; wherefore, we must look to the evils engendered by their religious and social system for the agents of this physical inferiority. In this system, the physiologist and moralist will not fail to detect the ample causes for a decay even so marked and melancholy. That this is not a mere fancy, or the result of prejudice, I may say, the same impression has been made upon all who have ever visited Salt Lake City, and published their opinions on the subject. Indeed, we find, in all the instincts and habits of these people, full confirmation of the physical facts above set forth. They are as gross and vulgar in all their tastes, thoughts and styles of expression as in their bodily appearance. More than half their language is made up of slang phrases, nor do they relish the efforts of their preachers, unless well interlarded with this style of speech. As a consequence, these men indulge freely in the most trivial, and, sometimes, in the most vulgar and blasphemous expressions, to the great delight and mental titillation of their hearers.

The Mormon, with few exceptions, is low-bred and vulgar. Dancing is his favorite amusement—forming, in fact, not only a pastime, but a part of his religious exercises. His conversation is of the most simple and commonplace character. His thoughts never soar above his amusements or domestic affairs. He deals in the gossip and scandal of his neighborhood. The Mormons, of both sexes, are an ill looking set, and when we have said they are frugal, industrious and content, we have enumerated about all the virtues they can claim, or that we can conscientiously concede to that wretched system of degradation known as Mormonism.

Under the polygamic system, the feeble virility of the male and the precocity of the female become notorious. The natural equilibrium of the sexes being disturbed, mischief of this kind must ensue; as a consequence, more than two thirds of the births are females, while the offspring, though numerous, are not long lived, the mortality in infantine life being very much greater than in monogamous society, and were it not for the European immigration, the increase of inhabitants would be actually less than in Gentile communities. The fecundity of the women is remarkable, as might be expected, considering that the husband cohabits with the wife only at such periods as are most favorable to impregnation.—*San Francisco Med. Press.*

